# Editorial: Chemical senses in health and disease

**DOI:** 10.3389/fncir.2026.1782975

**Published:** 2026-03-17

**Authors:** Carla Mucignat-Caretta, Sachiko Koyama, Vera V. Voznessenskaya

**Affiliations:** 1 Department of Molecular Medicine, University of Padova, Padua, Italy; 2 School of Medicine, Indiana University, Indianapolis, IN, United States; 3 Laboratory of Innovative Technologies, Severtsov Institute of Ecology and Evolution, Russian Academy of Sciences, Moscow, Russia

**Keywords:** chemical senses, chemosensory malfunction, COVID-19 pandemic, health sentinels, neurodegenerative disorders

The Research Topic “*Chemical senses in health and disease*” stemmed from the interest raised about the chemical senses during the COVID-19 pandemic. It appeared that SARS-CoV-2 infection could disturb both olfaction, ultimately leading to anosmia, and taste, leading to ageusia, yet also trigeminal chemesthesis could be affected (Parma et al., 2020).

The ability to detect molecules relevant for survival relies on a variety of different receptors, which differ in shape, binding ability and localization. Receptors are localized in specialized organs like the carotid bodies (Otlyga et al., 2025), which are sensitive to oxygen and carbon dioxide, pH, lactate and potassium, or even in the lungs (Kamra et al., 2025) or the gut (Raka et al., 2019). The receptor molecules pertain to different families and do not share common motifs or mechanism of action. By combining all the incoming information, our body continuously monitor both the external and internal environment, to best cope with possible perturbations, in order to maintain homeostasis with neuro-endocrine reflexes or even with behavioral adaptations. Since the olfactory circuits involve areas relevant for behavioral and emotional control, it is not surprising that chemosensory dysfunctions may be found in a variety of neurological and neuropsychiatric diseases (Brandt and Huppert, 2025). In addition, chemosensory dysfunction was suggested as one of the pillars for silent hypoxia (Machado and Paton, 2021; Caretta and Mucignat-Caretta, 2022) or gastrointestinal involvement in Covid-19 disease (Khreefa et al., 2023).

Considering the abovementioned widespread role of chemical receptors, the Topic was open to contribution from basic research, including biological studies in animal models of disease, to human studies, done on healthy volunteers, and to clinical science, with studies done on patients. The idea was to gather together contributions from areas of research that are often done apart and usually do not share discussion, like basic science on olfaction and clinical work on patients.

In the present Research Topic, four papers were included, two experimental and two with clinical relevance. Liu et al. present convincing evidence about the role of bitter taste receptors in the development of pneumonia due to *Staphylococcus aureus* infection in mice models. The TAS2R bitter taste receptors properly reside in the tongue, giving rise to conscious bitter perception, yet they may be found in a variety of other sites in the body. The Authors focus on the responses of lungs after infection of wild type mice, compared to five lines of mice lacking one or more bitter taste receptors. The genetic manipulations affected the responses to *S. aureus* infection, including the compensatory expression of other receptors and shifted toward an increased proliferation response, while weakening antimicrobial defense and impairing the recovery of lung tissue. The Authors also demonstrate that perturbation of the bitter taste signaling affects mTOR pathway and modulate the innate immune and inflammatory responses, leading to slowing of resolution processes in pneumonia. These data point to a possible druggable pathway, by acting on TAS2R, to improve recovery from bacterial pneumonia. This contribution has an interesting translational potential, since it demonstrates a relevant role for bitter taste receptors well beyond taste itself, and highlights a biological role for them in an organ, the lung, where detection of exogenous molecules may have a lifeguarding role.

In their brief report, Kosugi et al. highlight the role of carbonated water ingestion on cerebral blood flow in the frontal areas, as measured with near-infrared spectroscopy, a non-invasive powerful technique to study human cerebral blood flow. They start from the observation that ingestion of carbonated water stimulates swallowing, a complex function regulated by the brainstem. Moreover, it increases the blood flow in the middle cerebral artery, which supplies the temporal and frontal lobes. By comparing the 10 min before to the 10 min after ingestion, the Authors revealed that, despite the variability of the baseline signal, the oxyhemoglobin concentration increased in the left frontal region, an effect which was not apparent after still water ingestion. The location of the increase corresponds to the orbitofrontal cortex, which is the last station of the mesocortical rewarding system, a possible link with the pleasantness of sparkling water ingestion. This contribution underlines the strict links between ingestion of simple drinks (still or sparkling water) and hedonic evaluation done by the rewarding system, highlighting the interconnections between the different brain circuits that operate even in simple, everyday actions.

On a clinical framework, Saccardo et al. report the 4-years follow-up of patients that showed chemosensory dysfunctions, by comparing self-reported persisting symptoms to the psychophysical testing. Using the validated Sniffin' Sticks test and visual analog scales (VAS), the Authors report olfactory dysfunction in more than half of patients at the first timepoint, with a progressive recovery over the 4-year timeframe of the study, to leave 7.7% of patients still showing hyposmia at the last psychophysical test. A significant improvement was also perceived by patients over time, as demonstrated by VAS. The largest improvement took place during the first year, yet some improvements were still occurring afterwards, giving a chance for patients to persist in training. Recovery was positively linked to young age and olfactory training. This contribution opens an interesting clinical question, that is how long can we expect an improvement, after the onset of anosmia in human patients. The availability of a large number of patients whose pathophysiology was known, the onset of symptoms was monitored and the course of recovery was checked, represented a unique opportunity for clinicians. The finding that in most cases recovery improves, even if slowly, over 4 years is relevant not only for disease monitoring but also for clinical practice, where patients may be suggested to try to persist with olfactory training.

The article by Brosse et al. directly links the olfactory deficits specific for Parkinson's disease (PD) to distinctive structural features of the brain, that are not apparent in other forms of olfactory dysfunctions, not linked to PD. This is a very relevant clinical and scientific question, since olfactory dysfunction is common to a variety of diseases, yet it is very attractive for clinicians due the relative simplicity and non-invasiveness of its testing. In order to provide discriminative data relative to olfactory dysfunctions specific of Parkinson's disease, the authors collected structural data from the MRI scans of 15 PD patients and compared them to non-parkinsonian subjects, both functionally impaired in their olfaction, exploring the chemosensory areas for gray matter volumes and integrity of white matter tracts. By using specific processing methods and relevant statistics, the Authors detect only one volumetric difference, namely a significantly higher volume of the insula region of the left lobe in PD patients compared to controls. Interestingly no difference was detected in the white matter, pointing to the specificity of the gray matter effect. These results are of paramount importance for suggesting a clinical use of MRI of olfactory areas for early diagnosis of PD, an issue which is still open and in great need for new technical advances. The widespread use of structural MRI in clinical context further fosters the translational potential of this study.

In conclusion, the abovementioned papers contributed to improve our understanding of the role of the chemical senses as silent health guardians in health and disease. This Research Topic suggests that basic science may directly contribute to clinical science. Moreover, knowledge about chemical senses should not be limited to the traditional fields of olfaction and taste, suggesting new possible directions to follow in future research, by linking bench work to patients' care. Early detection of diseases may benefit from preclinical studies, that highlight the mechanisms of action of otherwise “ectopic” receptors. Also, studies on patients may contribute to better understanding the disease trajectory, to set up milestones for disease monitoring over time. They may also unravel new links between symptoms, and discover their biological substrate, contributing to the design of instruments for future, more sensitive tests for early detection of diseases. The puzzle of chemical sensing may extend well beyond what is identified in each single tile and may contribute to the design of the complex, intermingled machinery that allows us to stay alive.

## References

[B1] Brandt T. Huppert D. (2025). The mysterious sense of smell: evolution, historical perspectives, and neurological disorders. Front. Hum. Neurosci. 19:1588935. doi: 10.3389/fnhum.2025.1588935 40584825 PMC12203381

[B2] Caretta A. Mucignat-Caretta C. (2022). Not only COVID-19: involvement of multiple chemosensory systems in human diseases. Front. Neural Circuits 16:862005. doi: 10.3389/fncir.2022.862005 35547642 PMC9081982

[B3] Kamra K. Xia Z. Zucker I. H. Schultz H. Wang H. J. (2025). Chemoreflex function in pulmonary diseases - a review. J. Physiol. 603, 4461–4482. doi: 10.1113/JP286655 40744443 PMC12369309

[B4] Khreefa Z. Barbier M. T. Koksal A. R. Love G. Del Valle L. (2023). Pathogenesis and mechanisms of SARS-CoV-2 infection in the intestine, liver, and pancreas. Cells 12:262. doi: 10.3390/cells12020262 36672197 PMC9856332

[B5] Machado B. H. Paton J. F. R. (2021). Relevance of carotid bodies in COVID-19: a hypothetical viewpoint. Auton. Neurosci. 233:102810. doi: 10.1016/j.autneu.2021.102810 33894532 PMC8052558

[B6] Otlyga D. Otlyga E. Junemann O. Krivova Y. Saveliev S. (2025). The carotid body as part of a unified sympathoadrenal system of neural crest derivatives: insights from two centuries of research. Int. J. Mol. Sci. 26:11129. doi: 10.3390/ijms262211129 41303612 PMC12652928

[B7] Parma V. Ohla K. Veldhuizen M. G. Niv M. Y. Kelly C. E. Bakke A. J. . (2020). More than smell-COVID-19 is associated with severe impairment of smell, taste, and chemesthesis. Chem. Senses 45, 609–622. doi: 10.1093/chemse/bjaa041 32564071 PMC7337664

[B8] Raka F. Farr S. Kelly J. Stoianov A. Adeli K. (2019). Metabolic control via nutrient-sensing mechanisms: role of taste receptors and the gut-brain neuroendocrine axis. Am. J. Physiol. Endocrinol. Metab. 317, E559–E572. doi: 10.1152/ajpendo.00036.2019 31310579

